# Muscle satellite cell proliferation and association: new insights from myofiber time-lapse imaging

**DOI:** 10.1186/2044-5040-1-7

**Published:** 2011-02-02

**Authors:** Ashley L Siegel, Paige K Kuhlmann, DDW Cornelison

**Affiliations:** 1 Division of Biological Sciences, University of Missouri, Columbia, MO, USA; 2 Christopher S. Bond Life Sciences Center, University of Missouri, Columbia, MO, USA

## Abstract

**Background:**

As the resident stem cells of skeletal muscle, satellite cells are activated by extracellular cues associated with local damage. Once activated, satellite cells will re-enter the cell cycle to proliferate and supply a population of myoblasts, which will repair or replace damaged myofibers by differentiating and fusing either with an existing myofiber or with each other. There is also evidence that the orientation of cell division with respect to the myofiber may indicate or convey asymmetry in the two daughter cells. Our recent studies with time-lapse imaging of myofiber-associated satellite cells *in vitro *have yielded new data on the timing and orientation of satellite cell divisions, and revealed persistent differences in the behavior of daughter cells from planar versus vertical divisions.

**Results:**

We analyzed 244 individual fiber-associated satellite cells in time-lapse video from 24 to 48 hours after myofiber harvest. We found that initial cell division in fiber culture is not synchronous, although presumably all cells were activated by the initial trauma of harvest; that cell cycling time is significantly shorter than previously thought (as short as 4.8 hours, averaging 10 hours between the first and second divisions and eight hours between the second and third); and that timing of subsequent divisions is not strongly correlated with timing of the initial division. Approximately 65% of first and 80% of second cell divisions occur parallel to the axis of the myofiber, whereas the remainder occur outside the plane of the fiber surface (vertical division). We previously demonstrated that daughter cells frequently remain associated with each other after division or reassociate after a brief separation, and that unrelated cells may also associate for significant periods of time. We show in this paper that daughter cells resulting from a vertical division remain associated with one another several times longer than do daughters from a horizontal division. However, the total average time of association between sister cells is not significantly different from the total average time of association between unrelated cells.

**Conclusions:**

These longitudinal characterizations of satellite cell behavior shortly after activation provide new insights into cell proliferation and association as a function of relatedness, and indicate significant and consistent heterogeneity within the population based on these metrics.

## Background

Satellite cells are the resident stem cells of skeletal muscle; they are considered to be self-renewing, and serve to generate a population of differentiation-competent myoblasts that will participate as needed in muscle growth, repair and regeneration [1,2]. In mature muscle tissue, satellite cells occur as a small, dispersed population of mitotically and physiologically quiescent cells, marked by their expression of the transcription factor Pax7 [3] and several cell-surface markers, including CD34 [4], CXCR4 [5], syndecan-4 [6] and α7 integrin [7].

Because of their relative rarity and overall dispersion in the tissue, a useful method of visualizing satellite cells resident on relatively short muscles (either small muscles of larger animals such as rat, or muscles of a small animal such as mouse) is single-fiber isolation and culture [8-10]. Not only are satellite cells (once activated) clearly visible under the light microscope, but they can also be observed over time in relation to their parent myofiber and to other satellite cells resident on the same fiber. When fixed and stained with immune reagents, protein expression and localization can be observed in the context of the host myofiber and other satellite cells associated with the same fiber.

We have recently described a method of following fiber-associated satellite cells longitudinally over extended periods of time *in vitro*, using time-lapse microscopy [11]. This provides an advantage in characterizing satellite cell activity because we can directly visualize and follow individual satellite cells through multiple phases of activity, including exit from the basal lamina, proliferation, and movement along the myofiber. Although our previous work focused primarily on cell motility and the cellular and environmental factors required for efficient movement on the myofiber, a number of other activities were noted, including a much higher than expected degree of asynchrony in the timing of satellite cell divisions, and a surprising tendency for cells to both remain as cell doublets for extended periods of time after cell division and to associate as apparent doublets with unrelated cells. These behaviors would have a significant effect on interpretation and analysis of fixed and stained cell preparations, so we set out to tabulate and quantify these aspects of satellite cell activity after activation.

## Results

We analyzed 244 individual fiber-associated satellite cells over a 24-hour period, beginning at 24 hours after fiber harvest. All cells were considered 'activated' based on their rounded morphology, position outside the external lamina, and motility. For each cell, the time and axis of each cell division, the length of time the daughter cells remained associated, and any subsequent divisions or cell-cell associations (either with related or unrelated cells) were noted. All cultures contained fibroblast growth factor FGF2, a potent survival factor, but no other exogenous cytokines except those present in 15% horse serum. Cell by cell division data can be found in Table S1; Additional File 1. The data was extracted from movies available in Additional files 2, 3, 4, 5, 6, 7, 8 and 9.

### Timing of initial and subsequent proliferation

**Figure 1:**
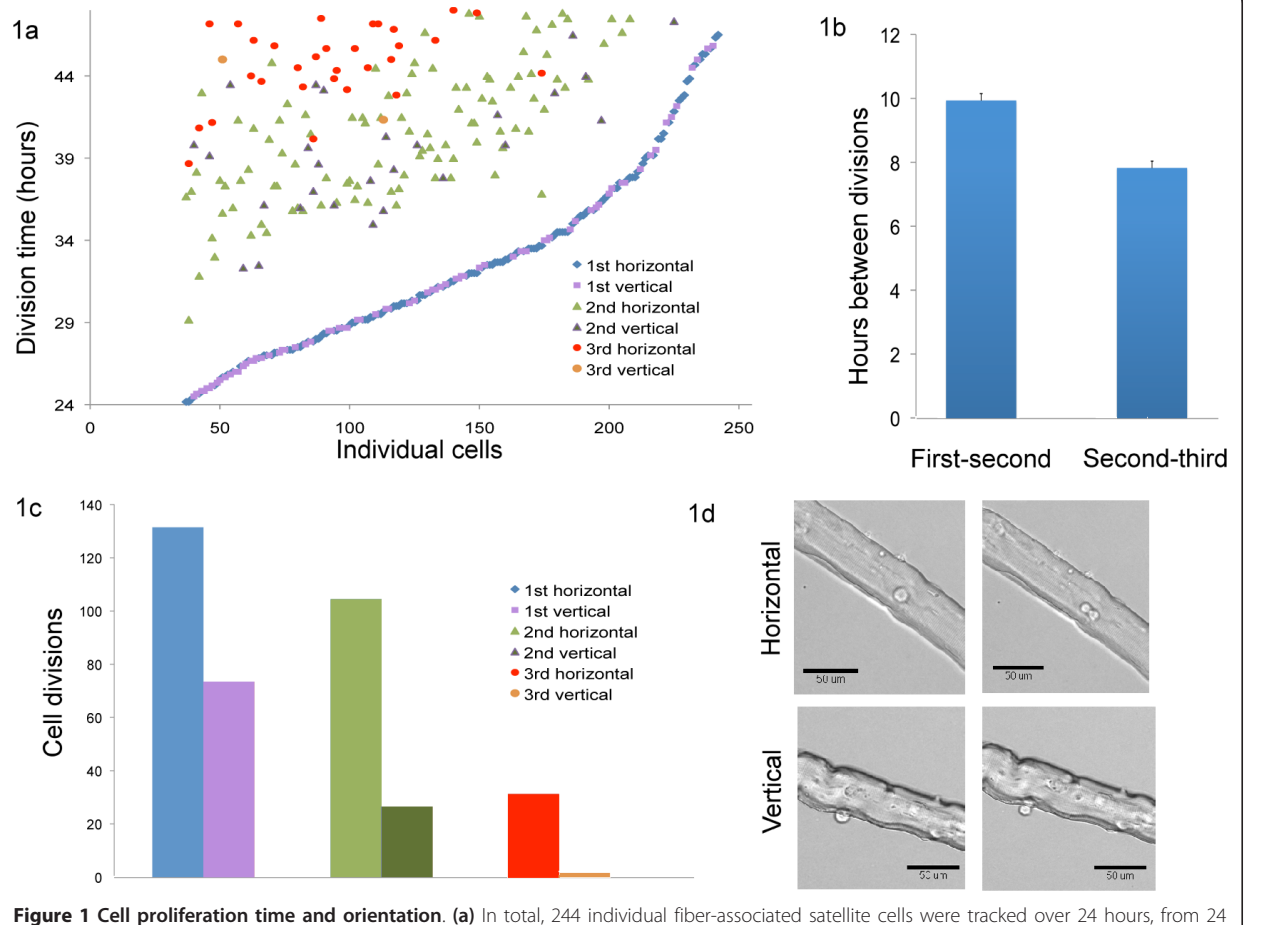
**Cell proliferation time and orientation**. **(a) **In total, 244 individual fiber-associated satellite cells were tracked over 24 hours, from 24 to 48 hours after activation by fiber harvest. Raw data are available as an Excel file (see Additional File 1, Table S1). For each cell, time and orientation of division (if any) were noted, and each division is represented by a marker coded to order (shape) and orientation (shade) of division. Individual cells were sorted from earliest to latest initial division time; 37 cells were not seen to divide at all (left end of *x *axis) although they were demonstrably activated, as evidenced by their morphology, position above the exterior lamina and motility. Subsequent divisions (if any) for each cell are also noted above the initial division at the time (hours) at which they occurred. Direction of division (horizontal = cells divide within the plane of the myofiber surface; vertical = axis of division is perpendicular to the myofiber) determines the marker fill color. **(b) **Average time between observed divisions (first to second, and second to third) in hours. Bar = SEM. **(c) **Quantification of total number of cells dividing either horizontally or vertically with respect to the plane of the myofiber for each observed division. (**d) **Sequential tagged image format (TIF) files (10 minutes apart) showing examples of (top) vertical and (bottom) horizontal division.

We found that, unlike results reported previously that were based on autoradiographic [8] or immunohistochemical [12] studies of cell cycle markers, neither the first division of satellite cells after harvest nor the subsequent divisions are synchronous when they are observed directly by time-lapse video. In our study, 16% of cells were never seen to divide; the remainder could undergo the first observed cell division at any time from 24 to 48 hours (Figure 1a). Subsequent divisions occurred in 65% of daughters anywhere from 5.1 to 17.8 hours after the first detected division (with an average cycling time of 10.0 hours), and 20% of cells divided a third time within the 24 hour observation period, 4.8 to 13.3 hours after the second division (with an average cycling time of 7.9 hours) (Figure 1b). These observed times are significantly shorter than the 16 to 18 hour cell cycle generally assumed for recently activated satellite cells [12].

### Orientation of division (horizontal versus vertical) with respect to the host myofiber

Among the 84% of cells that divided at least once during the 24 hours, 65% appeared to have an axis of division parallel to the myofiber axis (horizontal division) at the first division, whereas the other 35% had an axis of division perpendicular to the myofiber axis (vertical division) (Figure 1c, with representative cell divisions shown in Figure 1d). A small number of divisions were of indeterminate orientation and were not counted. Vertical divisions are therefore somewhat more prevalent in our dataset than observed in previous studies of planar versus apical division that used genetically marked satellite cells [13]. However, that study required a pair of fixed cells to be oriented vertically with respect to the fiber at the time of fixation, whereas in our time-lapse observations, we noted that frequently after a vertical division the cell doublet rapidly 'tipped' to leave both cells in contact with the myofiber (as would be observed in a horizontal division). The previous study was also only conducted at one timepoint (42 hours after harvest) and included determination of both symmetric and asymmetric marker gene expression. There may therefore have been some undersampling in that study, to the extent that similar cell activities were being measured. Interestingly, we found that vertical division with respect to the myofiber was not restricted to the first cell division, but also occurred in subsequent divisions: 20% of second divisions and 6% of third divisions observed in the 24 to 48 hour timeframe were also scored as occurring vertically (Figure 1a, b). To test whether the probability of dividing either vertically or horizontally was influenced by the orientation of the previous division, we performed a Pearson χ^2 ^test; the calculated χ^2 ^was 0.62, indicating that the null hypothesis is true and they are independent events. There also appeared to be no specific time period in which vertical divisions were more prevalent (Figure 1a).

### Association between related and non-related cells

**Figure 2:**
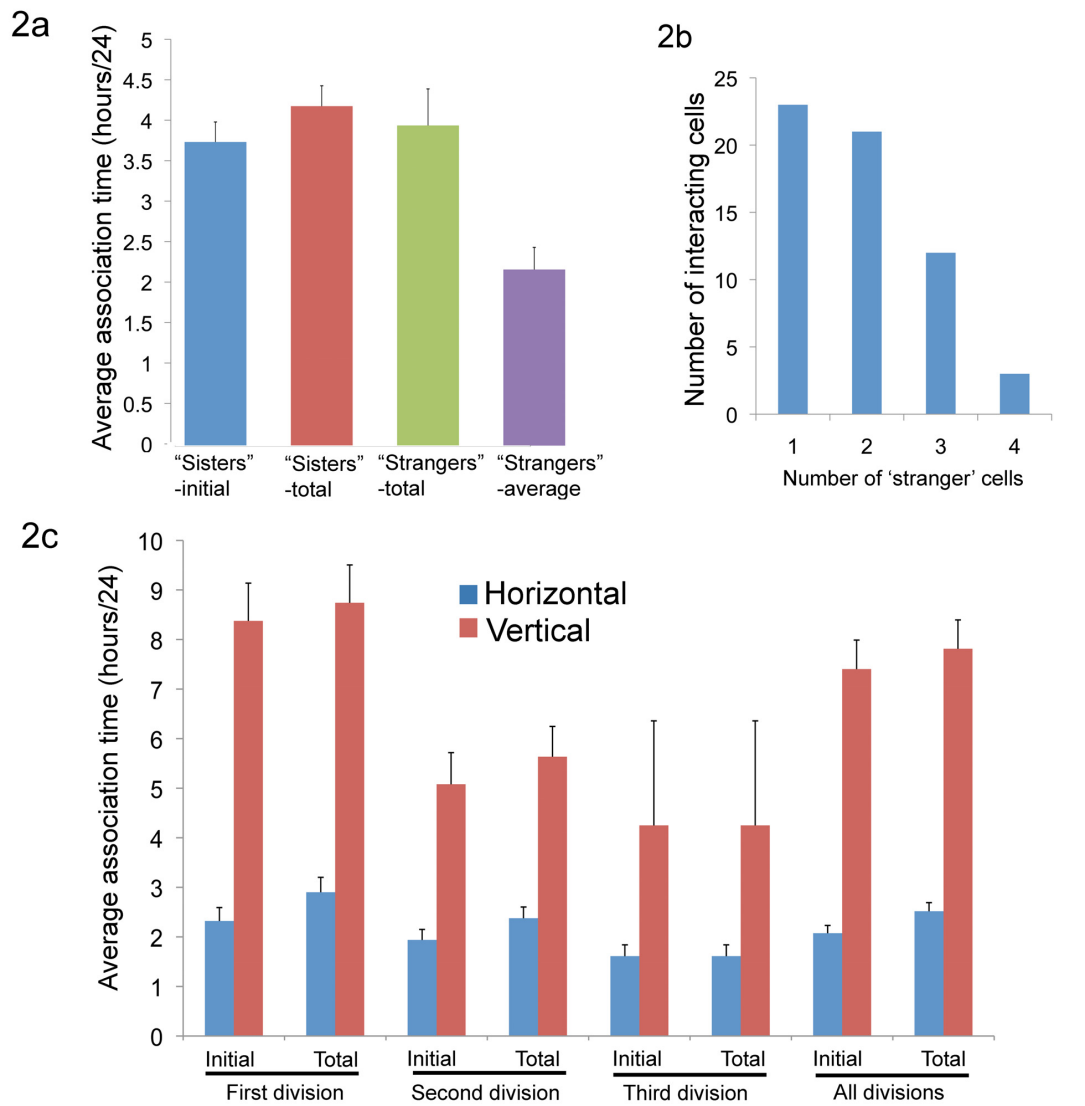
**Cell-cell association duration and distribution**. Of 244 tracked cells, 176 showed interactions with either a mitotic sister or an unrelated cell during the observation timeframe. **(a) **Average time (out of 24 hours) of associations between either 'sisters' (products of the same cell division) or 'strangers' (unrelated cells). Note that for sister cells, the associations immediately after cell division and total time including later reassociations are shown separately, whereas for unrelated cells, the total interaction time with any unrelated cell and the average association time with individual unrelated cells are shown separately. **(b) **Of the 176 cells, 66 interacted with unrelated satellite cells; most showed interactions with either one or two unrelated cells but some contacted up to four unrelated cells. **(c) **Average time (out of 24 hours) of association between sister cells that were the product of a horizontal division versus sisters from a vertical division, broken down by cell division number. Daughters of a vertical division were significantly more likely to remain associated for an extended period of time after cell division [*P *< 10^-8^, except for third-division cells, for which significance was poor because of the small sample size (only two cells dividing for the third time underwent vertical division)]. Daughters of the first observed division had an enhanced tendency to remain associated. Association time immediately after division and total time for all association between sister cells are shown separately.

A significant result of our previous time-lapse studies is that satellite cells occurring in closely associated doublets are not necessarily the product of a recent cell division: sister cells may remain associated for many hours after cell division, and motile satellite cells may encounter unrelated cells and appear as doublets in still images [11]. This complicates the analysis of proliferation and lineage history when evaluating fixed, stained populations of fiber-associated satellite cells, particularly with regard to establishing the relatedness of two heterogeneously staining cells. We found that there was only a slight preference for association with 'sister' rather than 'stranger' cells (Figure 2a), even though sister cells are in contact immediately after cell division and therefore would seem more likely to associate. Interestingly, those cells participating in non-sister cell associations could interact with up to four other non-sister cells in the 24 hour timeframe we examined (Figure 2b).

### Association between daughters of horizontal versus vertical divisions

Overall, the slight tendency to prefer 'sister' cells over 'stranger' cells in the population as a whole is due almost entirely to the significantly extended association times noted for daughters of vertical rather than horizontal divisions: the average association time between daughter cells from an initial vertical division was 8.4 hours, versus 2.3 hours for daughters from an initial horizontal division (Figure 2c). Sister cells that separated also occasionally reassociated and extended their total association time (Figure 2c). While both time of association for daughters of a vertical division and the fraction of cells dividing vertically decreased for second and third cell divisions, both of these phenomena were still prevalent. Averaged across all cells for the entire time period, cells with a history of vertical division were associated with sister cells for 7.8 hours, compared with 2.5 hours for the majority of cells resulting from exclusively horizontal divisions (Figure 2c).

## Discussion

We recently published a method by which single living myofiber explants together with their associated satellite cells are embedded in a collagen I gel, and the activity and motility of the satellite cells is captured by time-lapse video. It is important to emphasize that this system preserves some aspects of the *in vivo *influences available to the satellite cells but explicitly lacks others (including all secreted factors that would be produced by non-muscle cell types *in vivo*). The extracellular matrix (ECM), in particular, provides structural, adhesive and signaling cues to adherent cells, and probably influences multiple cellular activities.

The exterior lamina of the myofiber is composed primarily of collagen IV and laminin-2 (merosin) linked by entactin/nidogen [14]; the collagen forms the layer closest to the myofiber, whereas the laminin is more superficial. Therefore, when satellite cells reside beneath the basement membrane, they can encounter both collagen IV and laminin-2, whereas after they emerge from the sarcolemmal space, they encounter primarily laminin. When we stained for collagen IV and laminin after enzymatic myofiber harvest, both remained intact compared with non-enzymatically separated myofibers, except for localized areas of laminin depletion at sites of satellite cell exit (Siegel *et al*., manuscript in preparation). However, other ECM components that are not specifically associated with the myofiber lamina, including factors that have been shown to affect skeletal muscle regeneration, such as perlecan [15], would not be maintained in this system. Particularly in the context of vertical division, in which the 'top' cell in our culture would not be in contact with any physiological substrate besides its 'bottom' sister, this is a caveat to bear in mind.

Biophysical substrate properties have also been shown to affect cell activity: recent work from the Blau laboratory [16] has shown that satellite cell lineage choice and division behavior is regulated by substrate stiffness, with exposure to physical environments that are more similar to the elasticity of muscle tissue promoting enhanced stem-cell character and myogenic activity. The stiffness of the collagen I gel used in these experiments is calculated to be ~16 kPa [17], which is similar to the physiological stiffness of muscle *in vivo *(12 kPa) [16], thus the total surface of the satellite cells (on the myofiber and at the gel interface) is exposed to physiologically appropriate substrate pressure. Therefore, although the mechanical and structural components of this *ex vivo *system are more similar than other culture methods to a true *in vivo *environment, because it consists of only a single fiber embedded in a low-complexity matrix there are undoubtedly significant differences as well (including ones we are not yet aware of.)

We have previously derived quantitative data regarding cell velocity and directionality from sets of fiber-associated satellite cells viewed with time-lapse microscopy [11], but a significant amount of qualitative data remains to be extracted even from movies of experimentally untreated cells. The most surprising result from these analyses is that, contrary to most current thought in the field, initial cell division after myofiber harvest is quite asynchronous. We noted cell divisions occurring at all time points within the window we analyzed (24 to 48 hours after myofiber harvest). This could imply asynchrony in activation of individual satellite cells, followed by a consistent lag until initial division, or even asynchronous activation followed by a variable interval until the first division. However, this seems unlikely based on existing data regarding gene-expression changes associated with activation, and on our own observations of initiation of motility and subsequent exit from the sublaminar niche. In addition, whereas previous reports suggested a consistent cell-cycling time of about 17 to 18 hours [12] for satellite cells in fiber culture, we observed not only a much shorter average proliferation time (9.6 hours) but also a highly variable proliferation interval between the first, second and third divisions. It seems more likely that both the timing and length of the cell cycle are evidence of heterogeneity within the population for which we have not yet identified key mediators. Ideally, future experiments correlating expression of labeled candidate proteins with time-lapse analysis will provide insight into the molecular basis for this variation.

Such heterogeneity in individual satellite cell activity also appears to be supported by analysis of sister cell associations after either a vertical or horizontal division. Recently, compelling evidence has emerged that orientation of cell division with respect to the myofiber is predictive of both gene expression and stem-cell status [13,18]. We show here that orientation of division also correlates with subsequent cell behavior. Daughter cells resulting from cell divisions occurring in a vertical or planar division (which has been previously been associated with asymmetric stem-cell divisions, at a rate of ~10% of all divisions [13]) remained physically associated for several times longer (in some cases, for the remainder of the filming period after division, 23+ hours) than did daughters of a horizontal division. Although the reason for such prolonged contact is not yet clear, it is intriguing to speculate that extended contact-mediated signaling between a daughter cell that retains stem-cell characteristics and one that has committed to eventual myogenic differentiation (which would be expected based on genetic labeling studies [13,19]) may contribute to maintenance of the asymmetric fates. Contact-mediated reciprocal signaling, particularly through the Notch-Delta pathway, has been shown in other systems to reinforce asymmetric fates; further studies into paired-cell signaling may therefore be instructive.

## Conclusions

Time-lapse imaging is unlikely to replace analysis of fixed cell populations for many practical reasons. These include the time- and labor-intensive nature of the imaging and analysis, plus the difficulty of simultaneous visualization of molecular markers. The current study provides an analysis of general satellite cell morphometrics over a time period that is pertinent to the interpretation of fixed cell data. We reached four major conclusions: 1) Initial cell divisions following the activation of satellite cells by trauma signals produced during the myofiber harvest process are highly asynchronous, and the timing between subsequent cell divisions indicates a highly variable cell cycling time that is significantly shorter than previously reported. 2) Approximately 25% of all cell divisions occurring within the 24 to 48 hour time period are planar, with the axis of division perpendicular to the axis of the host myofiber. 3) When two cells are in close contact with one another, they are almost equally likely to be unrelated to each other as they are to be daughters of a single cell division. 4) Daughter cells resulting from a vertical division are 2 to 3 times more likely to be found associating with each other at any given point in time than are daughter cells of a horizontal division.

## Methods

### Myofiber harvest and culture

Viable myofiber explants from 80 to 130-day-old B6D2F1 female mice (Jackson) were prepared according to our published techniques **[**6,10,20]. Briefly, muscle was dissected from the hind limbs, carefully separated from associated tissues and digested in 400 U/ml collagenase type I (Worthington Biochemical, Lakewood NJ) diluted in Ham's F-12 medium (Invitrogen, Carlsbad CA). When single fibers are liberated, they are manually picked with a pipette and cultured at 37° and 5% CO_2 _in growth medium (Ham's F-12 (Invitrogen), 15% horse serum (Equitech Bio, Kerrville TX) and penicillin/streptomycin (Invitrogen, Carlsbad CA) supplemented with 0.5 nmol/L recombinant human FGF-2] for 24 hours before being transferred into 48-well plates for time-lapse analysis.

### Time-lapse capture

Myofibers were embedded in 200 μl per well of 2 mg/ml acid-extracted rat tail type I collagen [21] in growth medium in 48-well plates (Corning Costar). After polymerization, the wells were overlaid with growth medium containing 0.5 nmol/L FGF-2. Multiple fields under × 10 magnification were identified per well and marked for return. Images were collected automatically from each field every 10 minutes using IPLab (Scanalytics, Rockville MD) or MetaMorph (Molecular Devices, Sunnyvale CA).

### Post-imaging analysis

Stacked tagged image format (TIF) files generated by IPLab were imported into MetaMorph and arranged in sequential order. Cells were selected for analysis if they were 1) visible during the entire 24 hour imaging period, 2) distinguishable from other cells for the duration of the movie, and 3) associated with a viable myofiber for the duration of the movie. If a cell selected for tracking divided during the 24-hour collection period, one daughter cell was selected at random to continue the trace. For each cell, we noted the frame number(s) showing cell division, if any, the frame numbers during which the cell was in physical contact with another satellite cell, and the relationship between the two cells, if any. Frame numbers were converted to time after myofiber harvest for analysis.

## Competing interests

The authors declare that they have no competing interests.

## Authors' contributions

ALS and DDWC were responsible for myofiber harvest and culture. ALS performed the time-lapse imaging. PKK tabulated cell divisions and associations. DDWC conceived of the study, participated in its design and coordination, and wrote the manuscript. All authors read and approved the final manuscript.

## Supplementary Material

Additional file 1 **Table S1: Division timing and orientation data for all cells**. All 244 cells analyzed are listed by movie number (available as additional files 2, 3, 4, 5, 6, 7, 8, 9) and cell number. For each cell, time (in hours after myofiber harvest) and orientation of each division (H = horizontal; N = no division; V = Vertical) is noted. Click here for file

Additional file 2 **contains movies 1-15**. Click here for file

Additional file 3 **contains movies 16-30**. Click here for file

Additional file 4 **contains movies 31-45**. Click here for file

Additional file 5 **contains movies 46-60**. Click here for file

Additional file 6 **contains movies 61-75**. Click here for file

Additional file 7 **contains movies 76-90**. Click here for file

Additional file 8 **contains movies 91-105**. Click here for file

Additional file 9 **contains movies 106-121**. Click here for file
