# Supplementary material for: Muscle satellite cell proliferation and association: new insights from myofiber time-lapse imaging
Source: Skelet Muscle. 2011 Feb 2;1:7. doi: 10.1186/2044-5040-1-7 (PMC3157006; doi:10.1186/2044-5040-1-7)
Supplement: Additional file 2 — contains movies 1-15. [file 2044-5040-1-7-S2.ZIP › Movies1-15/Index.html]

Untitled Document


Supplemental Table 2  
Movie 1  
Movie 2  
Movie 3  
Movie 4   
Movie 5  
Movie 6  
Movie 7   
Movie 8  
Movie 9  
Movie 10  
Movie 11   
Movie 12  
Movie 13  
Movie 14  
Movie 15
