# Supplementary material for: Muscle satellite cell proliferation and association: new insights from myofiber time-lapse imaging
Source: Skelet Muscle. 2011 Feb 2;1:7. doi: 10.1186/2044-5040-1-7 (PMC3157006; doi:10.1186/2044-5040-1-7)
Supplement: Additional file 3 — contains movies 16-30. [file 2044-5040-1-7-S3.ZIP › Movies16-30/Index.html]

Untitled Document


Movie 16  
Movie 17  
Movie 18  
Movie 19  
Movie 20  
Movie 21  
Movie 22  
Movie 23  
Movie 24  
Movie 25  
Movie 26  
Movie 27  
Movie 28  
Movie 29  
Movie 30
