# Supplementary material for: Muscle satellite cell proliferation and association: new insights from myofiber time-lapse imaging
Source: Skelet Muscle. 2011 Feb 2;1:7. doi: 10.1186/2044-5040-1-7 (PMC3157006; doi:10.1186/2044-5040-1-7)
Supplement: Additional file 4 — contains movies 31-45. [file 2044-5040-1-7-S4.ZIP › Index.html]

Untitled Document


Movie 31  
Movie 32  
Movie 33  
Movie 34  
Movie 35  
Movie 36  
Movie 37  
Movie 38  
Movie 39  
Movie 40  
Movie 41  
Movie 42  
Movie 43  
Movie 44  
Movie 45
