# Supplementary material for: Muscle satellite cell proliferation and association: new insights from myofiber time-lapse imaging
Source: Skelet Muscle. 2011 Feb 2;1:7. doi: 10.1186/2044-5040-1-7 (PMC3157006; doi:10.1186/2044-5040-1-7)
Supplement: Additional file 5 — contains movies 46-60. [file 2044-5040-1-7-S5.ZIP › Index.html]

Untitled Document


Movie 46  
Movie 47  
Movie 48  
Movie 49  
Movie 50  
Movie 51  
Movie 52  
Movie 53  
Movie 54  
Movie 55  
Movie 56  
Movie 57  
Movie 58  
Movie 59  
Movie 60
