# Supplementary material for: Muscle satellite cell proliferation and association: new insights from myofiber time-lapse imaging
Source: Skelet Muscle. 2011 Feb 2;1:7. doi: 10.1186/2044-5040-1-7 (PMC3157006; doi:10.1186/2044-5040-1-7)
Supplement: Additional file 6 — contains movies 61-75. [file 2044-5040-1-7-S6.ZIP › Index.html]

Untitled Document


Movie 61  
Movie 62  
Movie 63  
Movie 64  
Movie 65  
Movie 66  
Movie 67  
Movie 68  
Movie 69  
Movie 70  
Movie 71  
Movie 72  
Movie 73  
Movie 74  
Movie 75
