# Supplementary material for: Muscle satellite cell proliferation and association: new insights from myofiber time-lapse imaging
Source: Skelet Muscle. 2011 Feb 2;1:7. doi: 10.1186/2044-5040-1-7 (PMC3157006; doi:10.1186/2044-5040-1-7)
Supplement: Additional file 7 — contains movies 76-90. [file 2044-5040-1-7-S7.ZIP › Index.html]

Untitled Document


Movie 76  
Movie 77  
Movie 78  
Movie 79  
Movie 80  
Movie 81  
Movie 82  
Movie 83  
Movie 84  
Movie 85  
Movie 86  
Movie 87  
Movie 88  
Movie 89  
Movie 90
