# Supplementary material for: Muscle satellite cell proliferation and association: new insights from myofiber time-lapse imaging
Source: Skelet Muscle. 2011 Feb 2;1:7. doi: 10.1186/2044-5040-1-7 (PMC3157006; doi:10.1186/2044-5040-1-7)
Supplement: Additional file 8 — contains movies 91-105. [file 2044-5040-1-7-S8.ZIP › Index.html]

Untitled Document


Movie 91  
Movie 92  
Movie 93  
Movie 94  
Movie 95  
Movie 96  
Movie 97  
Movie 98  
Movie 99  
Movie 100  
Movie 101  
Movie 102  
Movie 103  
Movie 104  
Movie 105
