# Supplementary material for: Muscle satellite cell proliferation and association: new insights from myofiber time-lapse imaging
Source: Skelet Muscle. 2011 Feb 2;1:7. doi: 10.1186/2044-5040-1-7 (PMC3157006; doi:10.1186/2044-5040-1-7)
Supplement: Additional file 9 — contains movies 106-121. [file 2044-5040-1-7-S9.ZIP › Index.html]

Untitled Document


Movie 106  
Movie 107  
Movie 108  
Movie 109  
Movie 110  
Movie 111  
Movie 112  
Movie 113  
Movie 114  
Movie 115  
Movie 116  
Movie 117  
Movie 118  
Movie 119  
Movie 120  
Movie 121
